# Area-level poverty and preterm birth risk: A population-based multilevel analysis

**DOI:** 10.1186/1471-2458-8-316

**Published:** 2008-09-15

**Authors:** Emily A DeFranco, Min Lian, Louis A Muglia, Mario Schootman

**Affiliations:** 1 Department of Obstetrics and Gynecology, Washington University School of Medicine, St. Louis, Missouri, USA; 2 Center for Preterm Birth Research, Washington University School of Medicine, St. Louis, Missouri, USA; 3 Department of Internal Medicine, Division of Health Behavior Research, Washington University School of Medicine, St. Louis, Missouri, USA; 4 Department of Pediatrics, Washington University School of Medicine, St. Louis, Missouri, USA

## Abstract

**Background:**

Preterm birth is a complex disease with etiologic influences from a variety of social, environmental, hormonal, genetic, and other factors. The purpose of this study was to utilize a large population-based birth registry to estimate the independent effect of county-level poverty on preterm birth risk. To accomplish this, we used a multilevel logistic regression approach to account for multiple co-existent individual-level variables and county-level poverty rate.

**Methods:**

Population-based study utilizing Missouri's birth certificate database (1989–1997). We conducted a multilevel logistic regression analysis to estimate the effect of county-level poverty on PTB risk. Of 634,994 births nested within 115 counties in Missouri, two levels were considered. Individual-level variables included demographics factors, prenatal care, health-related behavioral risk factors, and medical risk factors. The area-level variable included the percentage of the population within each county living below the poverty line (US census data, 1990). Counties were divided into quartiles of poverty; the first quartile (lowest rate of poverty) was the reference group.

**Results:**

PTB < 35 weeks occurred in 24,490 pregnancies (3.9%). The rate of PTB < 35 weeks was 2.8% in counties within the lowest quartile of poverty and increased through the 4^th ^quartile (4.9%), p < 0.0001. High county-level poverty was significantly associated with PTB risk. PTB risk (< 35 weeks) was increased for women who resided in counties within the highest quartile of poverty, adjusted odds ratio (_adj_OR) 1.18 (95% CI 1.03, 1.35), with a similar effect at earlier gestational ages (< 32 weeks), _adj_OR 1.27 (95% CI 1.06, 1.52).

**Conclusion:**

Women residing in socioeconomically deprived areas are at increased risk of preterm birth, above other underlying risk factors. Although the risk increase is modest, it affects a large number of pregnancies.

## Background

Preterm birth is a major public health burden whose prevalence continues to rise. The rate of preterm birth in the U.S. is 12.7%, the highest rate reported to date[1]. The financial burden of caring for infant survivors of preterm birth is substantial, with a lower-end estimate of approximately 26.2 billion dollars annually in the U.S.[2]. Preterm birth is a complex disease with etiologic influences from a variety of social, environmental, hormonal, genetic, and other factors[3]. Our limited understanding of the complex interactions among these factors contributes the lack of effective intervention strategies available to reduce the rate of preterm birth.

It has been hypothesized that the association between preterm parturition and individual-level socioeconomic deprivation is primarily accounted for by the co-existence of other significant risk factors such as medical comorbidities, lack of prenatal care, and adverse behaviors (smoking and alcohol use) which occur more commonly in women of lower socioeconomic status[4]. However, many prior studies have been limited by design constraints and the inability to thoroughly account for potential confounding factors.

Implementation of appropriate statistical methodologies, rigorous adherance to study design, and consideration of as many clinically important covariates as possible optimizes the capability to accurately quantify the independent association between area-level poverty and preterm birth risk. When taking into consideration that people who reside within the same area share common area-level neighborhood conditions, individual-based statistical models such as traditional logistic regression analyses are not optimal to determine the true strength of the association. Using multilevel logistic regression analysis has the advantage of allowing one to estimate not only the fixed effects of individual-level and area-level factors, but also the random effect of geographic variation of preterm birth between areas.

The purpose of this study was to utilize a large population-based birth registry to estimate the independent effect of county-level poverty on preterm birth risk. To accomplish this, we used a multilevel logistic regression approach to account for multiple co-existent individual-level variables and county-level poverty rate.

## Methods

A data set which included Missouri birth records from 1978–1998 was provided in a de-identified fashion for this analysis by the Missouri Department of Health and Senior Services. The study was considered exempt from review by the Missouri Department of Health and Senior Services IRB and the Human Subjects Committee of Washington University in St. Louis. This data set has been a rich source for the analysis of factors associated with birth timing [5-10].

A total of 1,577,082 births occurred in Missouri between 1978 and 1998. To optimize our capability to examine the influence of important individual factors, we limited our study to those births which occurred between 1989 and 1998 due to an unacceptable amount of missing demographic data for births that occurred before 1989 due to revisions in the birth certificate format in 1989 and improvements in edit and query systems after that date. Because deliveries which occur prior to 20 completed weeks of pregnancy are considered miscarriages rather than births, we limited our analysis to births recorded as having occurred at 20 weeks or greater. We excluded births resulting from a multifetal gestation, intrauterine fetal demise, or involving a major congenital malformation due to their know propensity to deliver preterm, potentially for mechanisms unrelated to the exposure we wished to evaluate. After these exclusions, the study population consisted of 675,044 births. We further limited our analysis to births occuring to mothers whose reported residence at the time of delivery was in the state of Missouri. There were 40,050 births in Missouri to mothers who resided in other states during the study period, yeilding a final population of 634,994 births available for analysis.

### Outcome measure

Preterm birth, as defined by the World Health Organization,[11] is a delivery which occurs at less than 37 completed weeks of gestation. We performed our primary analysis with preterm birth defined as less than 35 completed weeks in order to enrich for a population of deliveries which were truly preterm by avoiding births occuring at borderline gestational ages between 35 and 37 weeks, in an effort to minimize misclassification bias[12]. We defined early preterm birth as birth occuring prior to 32 completed weeks of gestation, because the risk of neonatal morbidity is higher for births of shorter gestations.

### Individual-level measures

We included the following individual-level measures: maternal age, maternal race (self-reported), maternal and paternal highest educational attainment, residence within city limits, birth sequence, marital status, presence of medical risk factors, marital status, indicators of low income (recipient of foodstamps, Medicaid, or WIC state-funded assistance), adequacy of prenatal care received, health-related behaviors (maternal tobacco or alcohol use), and presence of medical risk factors. Individual-level risk factors were selected from the data set based on clinical relevance and association with preterm birth[9].

Maternal and paternal education levels are recorded in the database in years of education completed, which we dichotomized as educational levels of < 12 years versus 12 years or more in order to identify those with less than a high school education. The variable of maternal education had minimal missing data, and paternal education was less complete with 22.6% missing data. A composite dichotomous variable of low socioeconomic status was created from the individual dichotomous variables of recipient of any of three state funded support programs (Medicaid, foodstamps, and Special Supplemental Nutrition Program for Women, Infants and Children [WIC]). A dichotomous variable of inadequate prenatal care was created from a continuous variable which indicated the month of pregnancy when prenatal care was initiated. Inadequate prenatal care was defined as having initiated prenatal care after 20 weeks of pregnancy, which is the latter half of pregnancy. A composite variable of heterogeneous medical risk factors indicated pregnancies complicated by anemia (hematocrit < 30% or hemoglobin < 10 gm/dL), maternal cardiac disease, acute or chronic lung disease, diabetes (insulin dependent), diabetes (other), genital herpes, hydramnios/oligohydramnios, hemoglobinopathy, chronic hypertension, pre-eclampsia, eclampsia, incompetent cervix, previous infant weighing > 4000 gm, previous preterm or small-for-gestational-age infant, maternal renal disease, Rh sensitization, or uterine bleeding.

### Area-level measure

Poverty rate was obtained from US census data (1990) and was defined as the percentage of the population falling below the US federal poverty line at the county level of the mother's reported residence as a measure of area socioeconomic position. The poverty rate is a measure that is robust across various diseases and levels of geography; it has a link to possible policy implications, and is comparable over time[13,14]. County-level poverty rates were divided into quartiles representing low poverty (first quartile, 0 – 6.86%), second quartile (6.89% – 11.91%), third quartile (11.92% – 14.49%) and high poverty (fourth quartile, ≥ 14.50%) to allow for nonlinear effects. The first quartile served as the reference group for comparison.

### Statistical analysis

We used multilevel logistic regression analysis to estimate the effect of county-level poverty on preterm birth risk. This analytic method can estimate not only fixed effects of individual-level and area-level covariates, but also the random effect of geographic variation of preterm birth across counties. When significant geographic variation exists, this indicates that preterm birth was not randomly distributed. The 634,994 births were nested within 115 counties in Missouri (the City of St. Louis acts politically as a county).

Data were analyzed with SAS GLIMMIX macro (version 9.1, SAS Institute Inc., Cary, NC). Findings with a p-value of < 0.05 were considered statistically significant.

We calculated median odds ratios (MOR) and interval odds ratios (IOR)[15,16] to estimate the effect of cluster-level (level 2) factors on preterm birth via several successive regression models (Models I through V). The methods for calculating MOR and IOR have been previously described and are directly comparable with the fixed-effects odds ratios[15,16]. MORs were calculated using the following equation:

$$MOR=\exp\left(Z_{0.75}*\sqrt{2*V_{C}}\right)$$

where *Z*_0.75 _is the 75th percentile of standard normal distribution, *V*_*C *_is the variance of PTB between counties, and exp(·) is the exponential function[15]. If the MOR is equal to 1, there is no variation between counties (no level 2 variation), but it is large if considerable intra-county variation exists.

80%-IORs were computed using the following equation:

$$\begin{array}{l} \begin{array}{cc} IOR_{Lower}=\exp\left(\beta +Z_{0.10}*\sqrt{2*V_{C}}\right), & \text{and} \end{array}\\ IOR_{Upper}=\exp\left(\beta +Z_{0.90}*\sqrt{2*V_{C}}\right) \end{array}$$

where *Z*_0.10 _and *Z*_0.90 _are the values of the standard normal distribution at the 10th and 90th percentiles, respectively. *β *is the regression coefficient of each category of county-level poverty rate. Larger geographic variation results in a broader range of the IOR. If the IOR does not cross the value of one, it suggests that county-level poverty substantially contributed to the geographic heterogeneity of PTB.

## Results

### Individual-level factors

The study population was comprised of 634,994 live births to mothers who resided in 115 counties in Missouri from 1989 – 1998 for an average of 5,522 births per county (range: 156 – 119,244). The racial distribution of the study cohort was 82.4% White, 16.1% Black, 0.3% Indian, 0.5% Asian/Pacific Islander, 0.6% other/unknown.

Table 1 demonstrates the mothers' baseline demographic, medical and obstetric characteristics according the quartile of poverty rate of the county in which they resided at the time of the birth. Women who resided in counties with higher rates of poverty were significantly younger, and more likely to be black, less likely to graduate from high school, to be unmarried, and be of low income. Likewise, parturiants residing in areas with higher county-level poverty were more likely to have inadequate prenatal care, smoke cigarettes, drink alcohol during pregnancy, and have at least one medical risk factor.

**Table 1:** Baseline characteristics of the study population according to county poverty rate

County Poverty Rate	Reference 1^st ^quartile	2^nd ^quartile	3^rd ^quartile	Highest Poverty 4^th ^quartile	p-value
Total	92,072	196,672	170,701	175,549	
Demographic factors					
Maternal age (mean ± SD)	27.0 ± 5.4	27.33 ± 5.8	25.4 ± 5.7	24.9 ± 5.8	< 0.0001
Reside inside city limits (n,%)	64,198 (69.1%)	121,346 (61.7%)	143,357 (84.0%)	124,625 (71.0%)	< 0.0001
Black race (n,%)	1,823 (2.0%)	29,384 (14.9%)	26,551 (15.5%)	47,140 (26.8%)	< 0.0001
Mother's education level < high school (n,%)	12,180 (13.2%)	26,930 (13.7%)	37,232 (21.8%)	51,609 (29.4%)	< 0.0001
Father's education level < high school (n,%)	7,697 (8.4%)	13,794 (7.0%)	19,384 (11.4%)	22,270 (12.7%)	< 0.0001
Married (n,%)	73,198 (79.5%)	147,841 (75.2%)	111,350 (65.2%)	102,479 (58.4%)	< 0.0001
Indicators of low SES * (n,%)	25,066 (27.2%)	64,428 (32.8%)	87,947 (51.5%)	111,913 (63.7%)	< 0.0001
Prenatal Care					
Inadequate prenatal care ** (n,%)	8,246 (9.0%)	21,285 (10.8%)	26,455 (15.5%)	38,045 (21.7%)	< 0.0001
Behaviors					
Maternal tobacco use (n,%)	21,084 (22.9%)	36,417 (18.5%)	40,774 (23.9%)	43,416 (24.7%)	< 0.0001
Maternal alcohol use (n,%)	1,703 (1.8%)	4,755 (2.4%)	4,003 (2.3%)	3,671 (2.1%)	< 0.0001
Medical Risk Factors					
Medical risk factors (n,%)	19,792 (21.5%)	37,235 (18.9%)	36,802 (21.6%)	40,055 (22.8%)	< 0.0001

* composite variable including recipient of state-funded Medicaid, foodstamps or WIC assistance

** prenatal care initiated after 20 weeks of pregnancy

### Preterm birth rate by county poverty rate

The overall rate of preterm birth < 35 weeks for the entire study population was 3.9%. The rate of preterm birth < 35 weeks was 2.8% for mothers who resided in counties within the lowest quartile of poverty (Table 2). The rate of preterm birth increased with increasing county poverty rate (2^nd ^quartile: 3.4%, 3^rd ^quartile: 3,9%, 4^th ^quartile: 4.9%), p < 0.0001. Similarly, early preterm births (< 32 weeks) occurred more commonly in mothers who resided in counties with higher poverty rates. The rate of early preterm birth nearly doubled from 1.0% in the 1^st ^quartile to 1.9% in the quartile with the highest poverty rate (p < 0.0001).

**Table 2:** Prevalence of preterm birth at less than 35 weeks and less than 32 weeks of gestation according to quartile of county-level poverty

County Poverty Rate	Reference 1^st ^quartile	2^nd ^quartile	3^rd ^quartile	Highest Poverty 4^th ^quartile	p value
Total	92,072	196,672	170,701	175,549	
PTB (< 35 wk)	2,551 (2.77%)	6,684 (3.40%)	6,671 (3.91%)	8,584 (4.89%)	< 0.0001
PTB (< 32 wk)	893 (0.97%)	2,521 (1.28%)	2,646 (1.55%)	3,402 (1.94%)	< 0.0001

Compared to births to mothers residing in counties with the lowest poverty rate (1^st ^quartile), the risk of preterm birth < 35 weeks increased with increasing rates of county-level poverty in univariate analysis (Table 3, Model I). This risk increase resulted in women in counties with the highest poverty rate being 1.30 times more likely to deliver preterm. The risk of early preterm birth was also significantly increased in mothers living in areas with the highest county-level poverty rate (OR 1.40; 95% CI 1.14, 1.72). This effect was similar when the study population was stratified by race. Both black and white mothers residing in counties with the highest poverty rate had an increased risk of preterm birth (Table 4).

**Table 3:** Multilevel logistic regression analysis results of preterm birth risk by quartile of county-level poverty, entire study population

	PTB (< 35 wk)			PTB (< 32 wk)		
	
	OR (95% CI)	IOR-80 *	MOR^†^	OR (95% CI)	IOR-80 *	MOR^†^
Model I: Poverty Only			1.21			1.22
2^nd ^Quartile	1.14 (0.94, 1.38)	0.79, 1.63		1.15 (0.92, 1.43)	0.79, 1.68	
3rd Quartile	1.28(1.06, 1.55)	0.89, 1.83		1.33 (1.07, 1.65)	0.91, 1.94	
4th Quartile	1.30(1.09, 1.56)	0.91, 1.87		1.40 (1.14, 1.72)	0.96, 2.05	
						
Model II: Model I + Demographic factors			1.10			1.07
2^nd ^Quartile	1.09 (0.97, 1.22)	0.91, 1.31		1.09 (0.97, 1.23)	0.96, 1.24	
3rd Quartile	1.18 (1.06, 1.32)	0.99, 1.42		1.24 (1.10, 1.39)	1.09, 1.41	
4th Quartile	1.15 (1.04, 1.28)	0.96, 1.39		1.25 (1.12, 1.41)	1.10, 1.43	
						
Model III: Model II + Prenatal Care			1.11			1.09
2^nd ^Quartile	1.09 (0.97, 1.22)	0.90, 1.32		1.09 (0.96, 1.25)	0.92, 1.30	
3rd Quartile	1.19 (1.06, 1.33)	0.98, 1.44		1.23 (1.08, 1.40)	1.04, 1.46	
4th Quartile	1.15 (1.03, 1.28)	0.95, 1.39		1.23 (1.08, 1.39)	1.03, 1.46	
						
Model IV: Model III + Behaviors			1.11			1.10
2^nd ^Quartile	1.09 (0.97, 1.23)	0.90, 1.33		1.10 (0.96, 1.25)	0.92, 1.31	
3rd Quartile	1.19 (1.06, 1.34)	0.99, 1.45		1.24 (1.08, 1.41)	1.04, 1.47	
4th Quartile	1.16 (1.04, 1.30)	0.96, 1.41		1.24 (1.09, 1.41)	1.04, 1.48	
						
Model V: Model III + Medical Risk Factors			1.14			1.18
2^nd ^Quartile	1.12 (0.97, 1.29)	0.87, 1.44		1.13 (0.94, 1.37)	0.83, 1.55	
3rd Quartile	1.23 (1.07, 1.42)	0.95, 1.59		1.28 (1.06, 1.55)	0.94, 1.75	
4th Quartile	1.18 (1.03, 1.35)	0.91, 1.52		1.27 (1.06, 1.52)	0.93, 1.73	

Refer to table 1 for a complete list of co-variates included in each model

*IOR-80 = 80% interval odds ratio

^†^MOR = median odds ratio

**Table 4:** Multilevel logistic regression analysis results of preterm birth risk by quartile of county-level poverty, stratified by maternal race

White	PTB (< 35 wk)			PTB (< 32 wk)		
	OR (95% CI)	IOR-80 *	MOR^†^	OR (95% CI)	IOR-80 *	MOR^†^

Model I: Poverty Only			1.12			1.07
2^nd ^Quartile	1.10 (0.97, 1.26)	0.88, 1.38		1.08 (0.96, 1.21)	0.95, 1.23	
3rd Quartile	1.25 (1.10 1.43)	1.00, 1.57		1.30 (1.16, 1.46)	1.14, 1.48	
4th Quartile	1.24 (1.10, 1.41)	0.99, 1.55		1.33 (1.19, 1.49)	1.17, 1.52	
						
Model II: Model I + Demographic factors			1.1			1.04
2^nd ^Quartile	1.09 (0.97, 1.21)	0.91, 1.30		1.06 (0.96, 1.17)	0.99, 1.14	
3rd Quartile	1.16 (1.04, 1.30)	0.97, 1.39		1.19 (1.08, 1.31)	1.11, 1.27	
4th Quartile	1.13 (1.02, 1.26)	0.95, 1.36		1.20 (1.09, 1.32)	1.12, 1.29	
						
Model III: Model II + Prenatal Care			1.1			1.07
2^nd ^Quartile	1.09 (0.97, 1.23)	0.90, 1.32		1.07 (0.95, 1.21)	0.94, 1.23	
3rd Quartile	1.17 (1.04, 1.31)	0.97, 1.41		1.18 (1.05, 1.33)	1.03, 1.34	
4th Quartile	1.13 (1.02, 1.26)	0.94, 1.37		1.18 (1.05, 1.32)	1.03, 1.35	
						
Model IV: Model III + Behaviors			1.11			1.07
2^nd ^Quartile	1.10 (0.98, 1.24)	0.91, 1.33		1.08 (0.96, 1.22)	0.95, 1.24	
3rd Quartile	1.18 (1.05, 1.33)	0.98, 1.43		1.19 (1.06, 1.34)	1.04, 1.36	
4th Quartile	1.15 (1.03, 1.28)	0.95, 1.39		1.20 (1.07, 1.34)	1.05, 1.37	
						
Model V: Model III + Medical Risk Factors			1.14			1.16
2^nd ^Quartile	1.12 (0.97, 1.30)	0.87, 1.45		1.12 (0.93, 1.34)	0.84, 1.49	
3rd Quartile	1.22 (1.05, 1.41)	0.94, 1.57		1.24 (1.03, 1.49)	0.93, 1.65	
4th Quartile	1.17 (1.02, 1.34)	0.91, 1.51		1.23 (1.04, 1.46)	0.92, 1.65	

Black	PTB (< 35 wk)			PTB (< 32 wk)		
	OR (95% CI)	IOR-80 *	MOR^†^	OR (95% CI)	IOR-80 *	MOR^†^

Model I: Poverty Only			1.2			1.22
2^nd ^Quartile	1.21 (0.88, 1.68)	0.86, 1.70		1.25 (0.82, 1.90)	0.85, 1.83	
3rd Quartile	1.26 (0.91, 1.76)	0.90, 1.77		1.39 (0.91, 2.14)	0.95, 2.04	
4th Quartile	1.46 (1.08, 1.98)	1.04, 2.05		1.33 (0.89, 1.97)	0.91, 1.94	
						
Model II: Model I + Demographic factors			1.11			1.19
2^nd ^Quartile	1.18 (0.90, 1.54)	0.96, 1.45		1.24 (0.83, 1.85)	0.90, 1.71	
3rd Quartile	1.18 (0.89, 1.55)	0.96, 1.45		1.34 (0.89, 2.02)	0.97, 1.86	
4th Quartile	1.33 (1.04, 1.72)	1.09, 1.64		0.87 (0.87, 1.85)	0.92, 1.76	
						
Model III: Model II + Prenatal Care			1.11			1.18
2^nd ^Quartile	1.18 (0.90, 1.54)	0.96, 1.44		1.23 (0.82, 1.82)	0.89, 1.68	
3rd Quartile	1.16 (0.88, 1.53)	0.95, 1.42		1.30 (1.87, 1.96)	0.95, 1.79	
4th Quartile	1.30 (1.01, 1.68)	1.06, 1.60		1.22 (0.83, 1.77)	0.89, 1.67	
						
Model IV: Model III + Behaviors			1.12			1.19
2^nd ^Quartile	1.16 (0.88, 1.52)	0.93, 1.44		1.21 (0.81, 1.81)	0.87, 1.68	
3rd Quartile	1.14 (0.86, 1.51)	0.92, 1.42		1.28 (0.85, 1.94)	0.93, 1.78	
4th Quartile	1.31 (1.01, 1.69)	1.05, 1.62		1.21 (0.83, 1.78)	0.88, 1.68	
						
Model V: Model III + Medical Risk Factors			1.16			1.29
2^nd ^Quartile	1.20 (0.89, 1.63)	0.91, 1.60		1.29 (0.80, 2.07)	0.79, 2.10	
3rd Quartile	1.16 (0.85 1.58)	0.87, 1.54		1.36 (0.84, 2.21)	0.84, 2.21	
4th Quartile	1.24 (0.93, 1.65)	0.93, 1.65		1.16 (0.74, 1.82)	0.71, 1.88	

Refer to table 1 for a complete list of co-variates included in each model

*IOR-80 = 80% interval odds ratio

^†^MOR = median odds ratio

### Multivariable modeling

When demographic factors are added to county-level poverty (Table 3, Model II), the odds ratio for preterm birth < 35 weeks of women residing in counties with the highest poverty rate was reduced from 1.30 to 1.15. Subsequent addition of other individual-level factors to the model, as demonstrated in models III, IV, and V, had minimal influence on the risk of preterm birth < 35 weeks (ORs ranging from 1.15–1.18) compared to the risk when only demographic factors are considered (4^th ^quartile, OR 1.15, 95% CI 1.09, 1.56).

When considering preterm births that occurred at earlier gestational ages (< 32 weeks of gestation), the risk increase associated with high county-level poverty was similarly influenced by individual-level factors. The unadjusted risk of early preterm birth with high county-level poverty (4^th ^quartile, OR: 1.40) was reduced to 1.0 when individual-level demographic factors were added to the model. The addition of other individual-level covariates such as prenatal care, behavioral risk factors, and medical risk factors only minimally influence the adjusted odds ratio (range 1.23 to 1.27).

Stratified analysis by race (black, white) demonstrated similar effect sizes when individual-level factors were added to the models (Table 4). Many more of the confidence intervals of risk in the analysis stratified by black race cross one than in the analysis by white race, but the effect sizes are very similar regardless of race.

There was a modest amount of geographic heterogeneity of PTB across counties in Missouri as measured by MOR and IOR. In all multilevel models, MOR values ranged from 1.07 to 1.22, and IOR-80s ranged from 0.79 to 2.05. As some IOR ranges did not contain one, this suggests that county-level poverty was an important area-level characteristic for geographic heterogeneity of PTB. But, other unmeasured area-level chacteristics could have also contributed to this geographic outcome variation.

## Discussion

In this study we found that women who gave birth in counties with the highest poverty rates were at increased risk of preterm birth at < 35 and < 32 weeks of gestation. Although these risks are modest, with odds ratio estimates of 1.18 and 1.27 respectively, the confidence intervals consistently excluded the value of one even after accounting for numerous individual demographic, obstetric, behavioral, and medical risk factors. These effects were similar regardless of maternal race. Modest increases in MOR and IOR values suggest that county-level poverty was an important area-level characteristic for geographic heterogeneity of PTB. But, other unmeasured area-level characteristics may also partly contribute to the geographic heterogeneity of preterm birth.

Other investigators have reported an association of individual and area-level socioeconomic deprivation with adverse perinatal outcomes, such as low birth weight and preterm birth[5,17-26]. Although numerous, these studies vary significantly with respect to sample size, study design, and methodologic strategies. Although women who are economically disadvantaged are disproportionately likely have co-existent risk factors for preterm birth, attempts to adjust for these factors in prior studies have not completely eliminated the modest association between poverty and prematurity. Our goal was to use optimal statistical techniques on a large-population based sample to describe the effect of regional poverty on prematurity. Due to the variety of information available, we were able to evaluate the effect of several partitions of clinically relevent social and medical factors on preterm birth while accounting for the random variation of prematurity across counties. Despite accounting for many sociodemographic, obstetric, medical, and other factors our results suggest that county environments may in fact independently influence birth outcomes.

The advantage of multilevel analytic approaches to health outcome research is that they enable us to evaluate and quantify the heterogeneity of the outcome across geographic areas. When minimal geographic heterogeneity exists, this implies high area-level correlation. When individuals within the population are similar and vary little with respect to health, this provides optimal conditions for regional prevention strategies[27]. Likewise, health outcomes such as preterm birth, which can be significantly influenced by behavior-related factors (smoking, alcohol use, receipt of prenatal care) may be less influenced by area-level factors than diseases with a long natural history such as atherosclerotic disorders. Also, considering that area-level effects are minimized as individuals move geographically,[15] it is unlikely that population mobility significantly accounted for our findings given the finite time of the gestational period. In the case of preterm birth, a serious complex disease process whose incidence continues to rise, refining our ability to identify at-risk populations in which to target preventative health care strategies has particular appeal.

The datasource used for this study has many advantages such as its large size and population-based nature. It includes a breadth of demographic information regarding the parents, detailed medical and obstetrical characteristics of the mother's antepartum and intrapartum course, as well as specific information regarding the status of the neonate at birth. Despite the extensive data available for analysis regarding each birth, there are some limitations to consider as well. One of the most commonly cited limitations of birth outcome research from vital statistics data is validity of the recorded gestational age[28]. In an effort to minimize the effect of gestational age estimation inaccuracies and potential misclassification bias,[12] we chose our primary outcome as birth prior to 35 weeks of gestation rather than the traditional definition of preterm birth, less than 37 weeks. The data set also had no information which could be used to reliably delineate spontaneous from medically indicated preterm births. Other limitations inherent to the datasource are more difficult to adjust such as the potential for undercoding or underreporting of obstetric and medical risk factors and errors in birth certificate data extraction and entry. A large amount of underreporting of important risk factors, if non-random, could have resulted in an increased effect size estimation of poverty on preterm birth risk due to the inability to adjust for the unreported risks in multivariable analyses. It is unlikely, though, that significant underreporting of the most important obstetric and medical risk factors occurred within the database period we evaluated as the relative prevalence of those included in our analyses are consistent with known rates elsewhere in the US[9]. On the other hand, many social and demographic variables are likely to be accurate, such as race, level of education, receipt of state-funded assistance programs, and county of residence.

Another limitation of the study is the lack of ability to account for other county-level factors which could have possibly influenced the association between poverty and preterm birth risk, such as regional crime rates, adverse environmental exposures, etc. We incorporated as many clinically importanty concomitant factors into our analysis as were available through the data source, but we acknowledge that other unmeasured and unknown potential co-existant risk factors could potentially exist.

In this study we chose counties as the geographic units of analysis. There are several reasons for this approach. US counties are the smallest geographical entity within a state with the social, political and legal responsibility for providing a broad range of social services and health care. Counties may qualify as communities to the extent that individuals and groups within them participate in community development by identifying county-wide problems, collecting health, social and environmental data, and by formulating and implementing specific public policy measures[29]. Counties are also the smallest geographical entity for which health, socioeconomic, and population statistics are consistently available over time. They are stable sociopolitical and geographic entities, but also provide an apppropriate socioeconomic, political, and community context within which many social and public health policies are formulated [28,30-32]. From a governmental perspective, using a county-based approach, rather than examining outcomes by zip code or census tracts, allows for organized implementation of healthcare policies and interventions.

In conclusion, county-level poverty was associated with an increased risk of preterm birth. Although the association is modest, even a modest increase in preterm birth risk as demonstrated in this study has the potential to have a robust effect given the large number of women residing in areas with relatively high poverty levels.

## Conclusion

Individual-level and area-level poverty have been associated in the past with an increased risk of preterm birth, but prior studies have been limited by design constraints and the inability to thoroughly account for potential confounding risks. We utilized a large population-based birth registry from the state of Missouri to perform a multi-level analysis of the association between poverty and preterm birth while accounting for many potential co-existent risks via optimal methodologic strategies. The findings from our study provide obstetrical care providers and health-care policy makers with important information regarding the prevalence of preterm birth in counties with high poverty and an accurate estimate of the effect of high area-level poverty on preterm birth risk.

## Competing interests

The authors declare that they have no competing interests.

## Authors' contributions

ED and LM developed the study hypothesis and along with MS contributed to the study design. ML and MS performed the data analysis and assisted the other coauthors with its interpretation. ED wrote the first draft of the manuscript. All authors contributed to revision and preparation of the final manuscript.

## Pre-publication history

The pre-publication history for this paper can be accessed here:



## References

[B1] Hamilton BE, Martin JA, Ventura SJ (2006). Births: preliminary data for 2005. Natl Vital Stat Rep.

[B2] (2006). Committee on Understanding Premature Birth and Assuring Healthy Outcomes. Preterm Birth: Causes, Consequences, and Prevention.

[B3] DeFranco E, Teramo K, Muglia L (2007). Genetic influences on preterm birth. Seminars in reproductive medicine.

[B4] Goldenberg R, Nagahawatte NT (2008). Poverty, Maternal Health and Adverse Pregnancy Outcomes. Ann N Y Acad Sci.

[B5] Ananth CV, Getahun D, Peltier MR, Salihu HM, Vintzileos AM (2006). Recurrence of spontaneous versus medically indicated preterm birth. American journal of obstetrics and gynecology.

[B6] Ananth CV, Peltier MR, Getahun D, Kirby RS, Vintzileos AM (2007). Primiparity: an 'intermediate' risk group for spontaneous and medically indicated preterm birth. J Matern Fetal Neonatal Med.

[B7] Ananth CV, Vintzileos AM (2006). Maternal-fetal conditions necessitating a medical intervention resulting in preterm birth. American journal of obstetrics and gynecology.

[B8] DeFranco EA, Stamilio DM, Boslaugh SE, Gross GA, Muglia LJ (2007). A short interpregnancy interval is a risk factor for preterm birth and its recurrence. American journal of obstetrics and gynecology.

[B9] Kistka ZA, Palomar L, Lee KA, Boslaugh SE, Wangler MF, Cole FS, DeBaun MR, Muglia LJ (2007). Racial disparity in the frequency of recurrence of preterm birth. American journal of obstetrics and gynecology.

[B10] Palomar L, DeFranco EA, Lee KA, Allsworth JE, Muglia LJ (2007). Paternal race is a risk factor for preterm birth. American journal of obstetrics and gynecology.

[B11] WHO (1992). World Health Organization (WHO). International statistical classification of diseases and related health problems (revision 10; vols 1 and 2; ICD-10).

[B12] Balchin I, Whittaker JC, Steer PJ, Lamont RF (2004). Are reported preterm birth rates reliable? An analysis of interhospital differences in the calculation of the weeks of gestation at delivery and preterm birth rate. Bjog.

[B13] Dibben C, Sigala M, Macfarlane A (2006). Area deprivation, individual factors and low birth weight in England: is there evidence of an "area effect"?. Journal of epidemiology and community health.

[B14] Krieger N, Chen JT, Waterman PD, Soobader MJ, Subramanian SV, Carson R (2002). Geocoding and monitoring of US socioeconomic inequalities in mortality and cancer incidence: does the choice of area-based measure and geographic level matter?: the Public Health Disparities Geocoding Project. American journal of epidemiology.

[B15] Larsen K, Merlo J (2005). Appropriate assessment of neighborhood effects on individual health: integrating random and fixed effects in multilevel logistic regression. American journal of epidemiology.

[B16] Larsen K, Petersen JH, Budtz-Jorgensen E, Endahl L (2000). Interpreting parameters in the logistic regression model with random effects. Biometrics.

[B17] Kogan MD (1995). Social causes of low birth weight. Journal of the Royal Society of Medicine.

[B18] Kramer MS (1987). Determinants of low birth weight: methodological assessment and meta-analysis. Bulletin of the World Health Organization.

[B19] Parker JD, Schoendorf KC, Kiely JL (1994). Associations between measures of socioeconomic status and low birth weight, small for gestational age, and premature delivery in the United States. Annals of epidemiology.

[B20] Pearl M, Braveman P, Abrams B (2001). The relationship of neighborhood socioeconomic characteristics to birthweight among 5 ethnic groups in California. American journal of public health.

[B21] Rauh VA, Andrews HF, Garfinkel RS (2001). The contribution of maternal age to racial disparities in birthweight: a multilevel perspective. American journal of public health.

[B22] Roberts EM (1997). Neighborhood social environments and the distribution of low birthweight in Chicago. American journal of public health.

[B23] Vinikoor LC, Kaufman JS, Maclehose RF, Laraia BA (2008). Effects of racial density and income incongruity on pregnancy outcomes in less segregated communities. Soc Sci Med.

[B24] Esperat C, Du F, Yan Z, Owen D (2007). Health behaviors of low-income pregnant minority women. Western journal of nursing research.

[B25] Smith LK, Draper ES, Manktelow BN, Field DJ (2007). Deprivation and infection among spontaneous very preterm births. Obstetrics and gynecology.

[B26] O'Campo P, Burke JG, Culhane J, Elo IT, Eyster J, Holzman C, Messer LC, Kaufman JS, Laraia BA (2008). Neighborhood Deprivation and Preterm Birth among Non-Hispanic Black and White Women in Eight Geographic Areas in the United States. American journal of epidemiology.

[B27] Merlo J (2003). Multilevel analytical approaches in social epidemiology: measures of health variation compared with traditional measures of association. Journal of epidemiology and community health.

[B28] Alexander GR (1997). The accurate measurement of gestational age–a critical step toward improving fetal death reporting and perinatal health. American journal of public health.

[B29] Warren R (1978). The community in America.

[B30] Singh G (2003). Area deprivation and widening inequalities in US mortality, 1969–1998. American journal of public health.

[B31] Singh G, Siahpush M (2002). Increasing inequalities in all-cause and cardiovascular mortality among US adults aged 25–64 by area socioeconomic status, 1969–1988. International Journal of Epidemiology.

[B32] Singh GK, Miller BA, Hankey BF, Feuer EJ, Pickle LW (2002). Changing area socioeconomic patterns in U.S. cancer mortality, 1950–1998: Part I–All cancers among men. J Natl Cancer Inst.

